# The effectiveness of the Safety in Interventional Radiology (SIR) Shield in reducing droplet transmission and its effect on image quality and radiation dose

**DOI:** 10.1259/bjr.20210835

**Published:** 2021-10-21

**Authors:** Shao Jin Ong, Gopinathan Anil, Koon Liang Chia, Deborah Khoo, Joseph KT Lee, Priscilla XH Chen, Teddy M Nares, Calvin J Koh, Peijing Su, Cunli Yang, Pavel Singh, Prapul C Rajendran, Timothy Fotheringham, Swee T Quek, Ian Renfrew

**Affiliations:** 1 Department of Diagnostic Imaging, National University Hospital Singapore, Singapore, Singapore; 2 Department of Diagnostic Radiology, Yong Loo Lin School of Medicine, National University of Singapore, Singapore, Singapore; 3 Department of Anaesthesia, National University Hospital Singapore, Singapore, Singapore; 4 Department of Radiology, The University of North Carolina, Chapel Hill, North Carolina, USA; 5 Dept of Medicine, Yong Loo Lin School of Medicine, National University of Singapore, Singapore, Singapore; 6 Division of Gastroenterology and Hepatology, National University Hospital Singapore, Singapore, Singapore; 7 Department of Medicine, National University Hospital Singapore, Singapore, Singapore; 8 Department of Radiology, Royal London Hospital, Barts Health NHS Trust, London, United Kingdom

## Abstract

**Objective:**

To evaluate the efficacy of a barrier shield in reducing droplet transmission and its effect on image quality and radiation dose in an interventional suite.

**Methods:**

A human cough droplet visualisation model in a supine position was developed to assess efficacy of barrier shield in reducing environmental contamination. Its effect on image quality (resolution and contrast) was evaluated via image quality test phantom. Changes in the radiation dose to patient post-shield utilisation was measured.

**Results:**

Use of the shield prevented escape of visible fluorescent cough droplets from the containment area. No subjective change in line-pair resolution was observed. No significant difference in contrast-to-noise ratio was measured. Radiation dosage to patient was increased; this is predominantly attributed to the increased air gap and not the physical properties of the shield.

**Conclusion:**

Use of the barrier shield provided an effective added layer of personal protection in the interventional radiology theatre for aerosol generating procedures.

**Advances in knowledge:**

This is the first time a human supine cough droplet visualisation has been developed. While multiple types of barrier shields have been described, this is the first systematic practical evaluation of a barrier shield designed for use in the interventional radiology theatre.

## Introduction

With the outlook of the COVID-19 pandemic stretching into the foreseeable future and multiple waves with new variants of concern occurring around the world, hospitals need to adapt to the “new normal”.^1^ COVID-19 is known to spread predominantly via droplets with increasing concerns of aerosol transmission. A barrier shield which we have named Safety in Interventional Radiology (SIR) Aerosol Generating Procedure (AGP) Shield was developed rapidly together with the UK Manufacturing Technology Centre engineers, working in collaboration with Rolls-Royce^2^ to reduce the risk of regional droplet spread from aerosol generating procedures.

**Figure 1.**
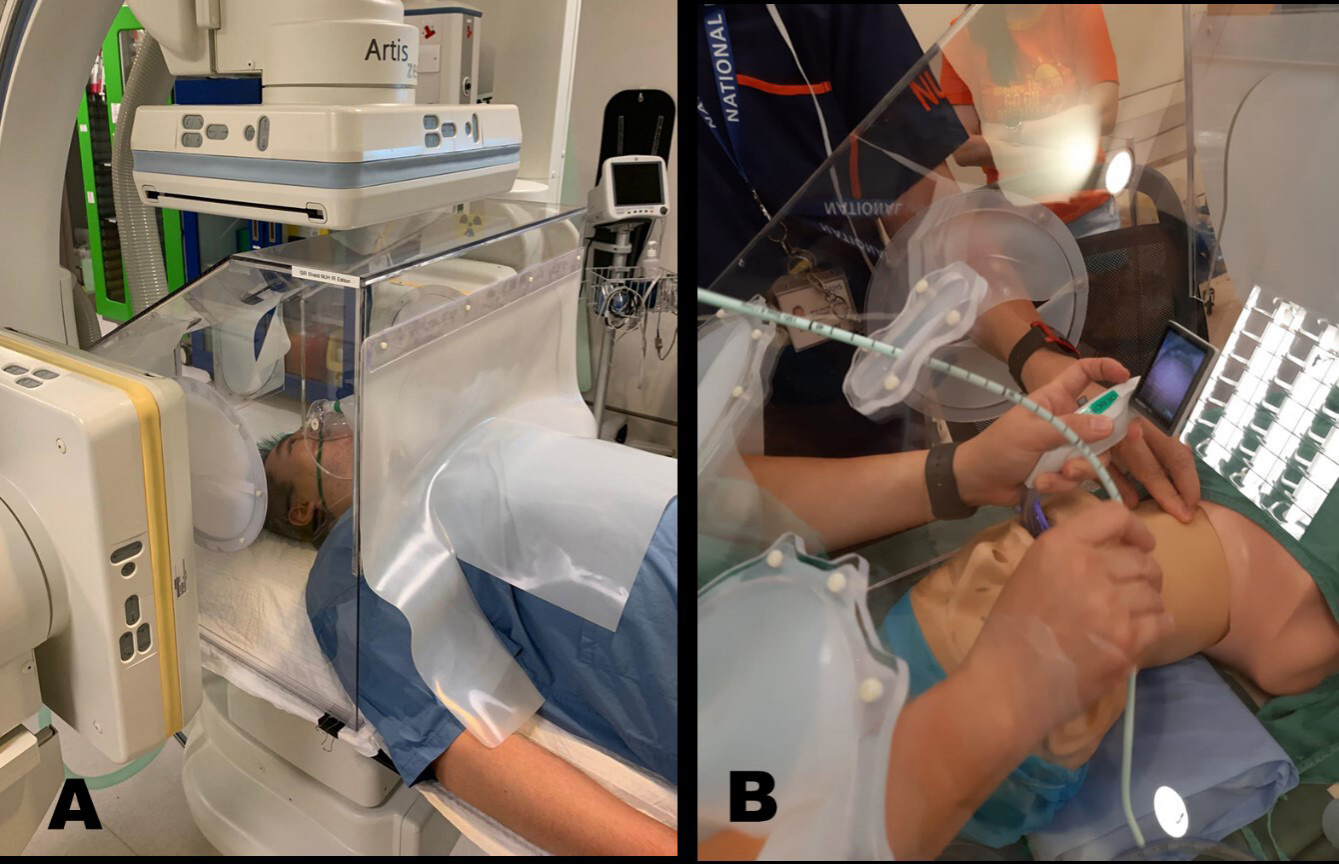
SIR AGP Shield. (**a**) Demonstration of use within a bi-planar Interventional radiology theatre. (**b**) Side-holes with overlapping silicone flaps for assistant access in simulated intubation–extubation procedures and a cranial access port for intubation bougie and endotracheal tube access. AGP, Aerosol Generating Procedure; SIR, Safety in Interventional Radiology.

The evaluated first-generation Shield was fabricated with 5 mm polycarbonate, 1.5 mm silicone sheets, plastic screws and chloroform as bonding agent based on the technical design pack.^3^ It is a polygonal box with partially overlapping silicone flaps access on two sides of the box to facilitate access and dexterity for procedures with a silicone curtain on the patient side (Figure 1a). A small silicone flap window on the cranial aspect was for intubation bougie and endotracheal tube access (Figure 1b).

The calculated functional volume of the Shield was approximately 195 l. With a standard hospital wall vacuum providing an air flow rate of at least 40 l/min, this gives just over 12 air changes per hour (ACH).

We undertook this study to evaluate the effectiveness of the Shield in reducing droplet transmission, its effect on image quality, and radiation dose to the patient.

## Methods and materials

### Human cough droplet visualisation model

Edible fluorescent fluid was derived by dissolving vitamin B complex (Berroca, Bayer, Leverkusen, Germany) in quinine-based soda (Premium Indian Tonic Water, Fever Tree, London, UK). Marking sheets were laid out in a 10 × 10m square and taped to the ground. Healthy volunteers gargled and ingested 20 ml of fluid, and coughed three times with their head centred within the square in a closed room. Droplets were allowed to settle for 30 min prior to evaluation. Three researchers assessed the splatter pattern and droplet range under ultraviolet-A light. Droplets were only included if there was consensus among all assessors.

### Optical clarity of SIR AGP Shield

A polycarbonate test plate (identical to the construction material of the Shield) was separated into sections and subjected to repeated cleaning using surface disinfecting products: Ammonia-based Mikrozid Sensitive wipes (Schulke, Norderstedt, Germany), Virusolve+^®^ Sporicidal Wipes (Amity International, Barnsley, UK) and 70% isopropyl alcohol wipes. To simulate repeated clinical use, each section was wiped once, left to dry and repeated 200 times. All sections were cleaned using a microfiber cloth before and after wiping prior to evaluation.

A light transmission test was carried out using a calibrated light detector (RaySafe Xi, Unfors RaySafe AB, Sweden). Each section of each test plate was inserted between a light box and the detector, and corresponding light intensity measurements were recorded. This process was performed 10 times to obtain ten sets of reference and intensity readings, before and after 200 cycles of cleaning.

### Effect of SIR AGP Shield on image quality

Slabs of 25 × 25 mm polymethyl methacrylate (PMMA) to a total thickness of 128 mm were placed on the couch of a Siemens Artis Zee Biplane system to simulate an adult patient. An image quality test phantom consisting of spatial resolution line-pairs ranging from 0.50 to 6.00 line-pairs per mm and 24 contrast dots was placed atop the PMMA slabs.

The limiting spatial resolution was evaluated subjectively with 12 readers evaluating 10 blinded images of the phantom taken in five different settings with and without the Shield with the X-ray tube and the image intensifier in the same position. Images were exported and attached onto a PowerPoint slide show to standardise viewing and restrict image manipulation.

Image contrast was evaluated by calculating the contrast-to-noise (CNR) ratio using the ImageJ software (Shareware, National Institutes of Health). The Mann–Whitney *U* test was carried out to investigate the impact of the SIR shield on image contrast.

### Effect of SIR AGP Shield on radiation dose

A calibrated solid-state dosemeter (RaySafe Xi, Unfors RaySafe AB, Sweden) was placed under the PMMA slabs, positioned at the edge of the field-of-view, and oriented perpendicular to the anode-cathode axis of the tube. Three positioning protocols were defined: native, elevated without Shield, and elevated with Shield. Native positioning simulated a routine exam by setting the table height to 98 cm and the detector height to 124 cm. Elevated positionings simulated use of the Shield with table and detector heights of 82 cm and 143 cm without and with the Shield in place.

In single plane AP mode, five exposures were made based on machine default medium-sized patient settings to simulate clinical use: (A) 60 s fluoroscopy, (B) 20 s Digital Subtraction Angiography (DSA) on two frames per second (FPS), (C) 20 s DSA on 3 FPS, (D) 20 s DSA on 6 FPS and (E) single shot acquisition.

All K_a,i_ (Incident Air Kerma) readings from the dosemeter, and all K_a,r_ (Air Kerma at the Interventional Reference Point) and P_KA_ (Kerma–Area Product) measurements from the Artis system were recorded.

## Results

### Human cough droplet visualisation model

**Figure 2.**
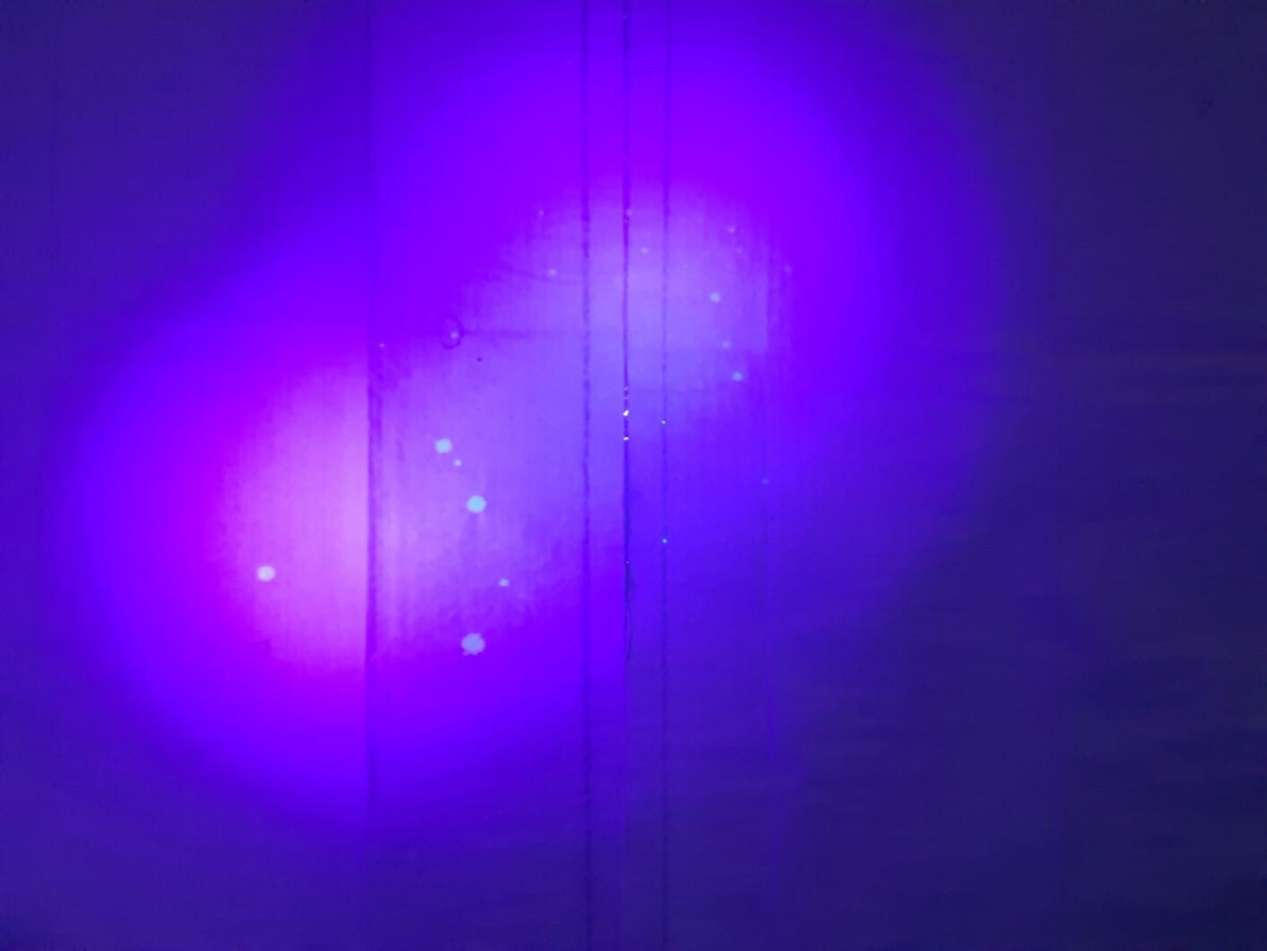
Fluorescent cough droplets. Edible fluorescent fluid was gargled and ingested by healthy human volunteers prior to coughing supine in a dark room. Cough droplet splatter was visualised with ultraviolet-A light.

Four male volunteers (aged 35–41) took part in the cough visualisation experiment. There was good visualisation of the fluorescent droplets with three-person consensus on the localised and measurable droplets (Figure 2). Majority of the fluid droplets were centred around the head within an 80 cm radius. Average maximal distance travelled by the visualised cough droplet on the same horizontal plane was 158 cm (max 201 cm, min 114 cm, SEM 18.7 cm).

With the introduction of the AGP barrier shield, no fluorescent droplets were visualised on the marking sheets outside the containment area of the Shield.

### Optical clarity of SIR AGP Shield

**Figure 3.**
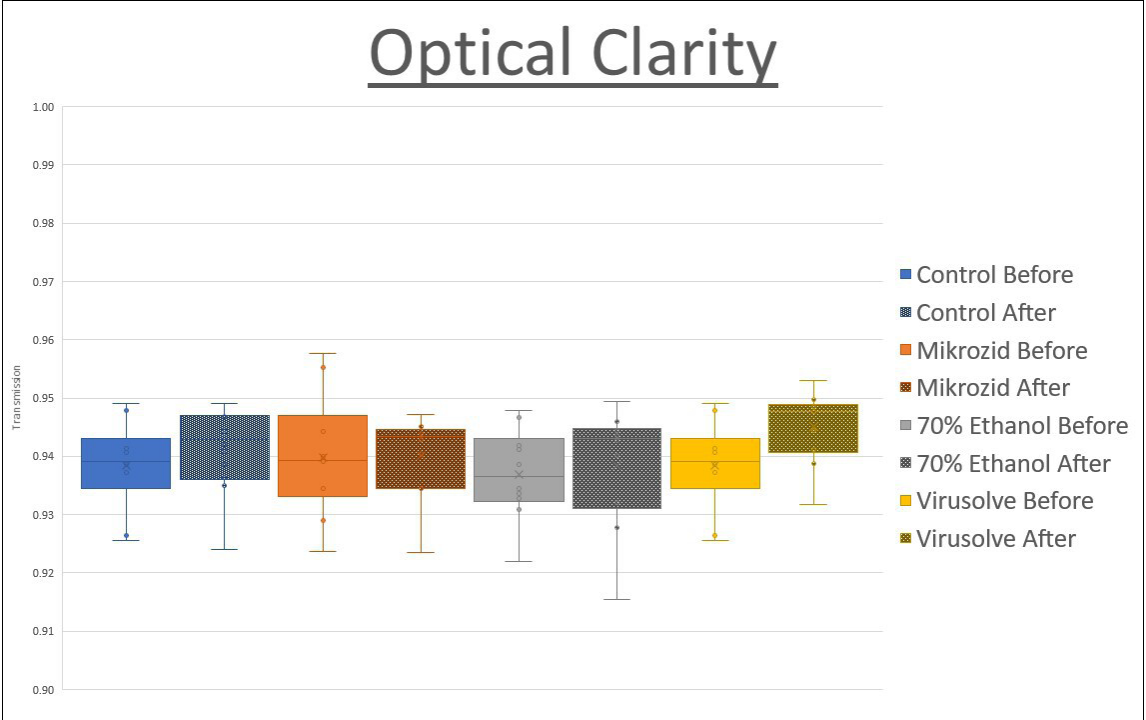
Optical clarity pre- and post-200 cycles of cleaning with hospital disinfectants. Optical clarity of 5 mm polycarbonate was measured pre- and post-200 cycles of cleaning with Ammonia-based Mikrozid Sensitive wipes, Virusolve+^®^ Sporicidal Wipes and 70% isopropyl alcohol wipes. No significant difference was observed between the optical clarity pre- and post-200 cycles of cleaning with either of the disinfectants.

The light transmission was calculated by taking the average ratio of the detected light intensity of each section to its corresponding reference. Ammonia-based Mikrozid stayed constant at 0.940 to 0.940, Virusolve increased from 0.939 to 0.945 and 70% ethanol had a slight increase from 0.937 to 0.939. A two-tailed Mann–Whitney *U* test was carried out at the 5% level of significance. No significant difference was found between the light transmissions, both before and after using the disinfection agents (Figure 3).

### Effect of SIR AGP Shield on image quality

The highest visualised resolution reported was three lines per mm on DSA and single image acquisition, and the lowest was at 1.25 lines per mm on fluoroscopy. 10 out of 12 reviewers reported a higher line-pair count between the DSA and single shot acquisition than fluoroscopy, while the last two reviewers did not identify any change in all the 10 images. Among the 60 sets of paired images, 58 sets were reported to have identical line-pair resolution with and without the Shield (Table 1).

**Table 1.** Line pairs per mm visible on fluoroscopy, 2 FPS DSA, 3FPS DSA, 6FPS DSA and single shot acquisition with and without the shield

Line pairs per mm	Fluoroscopy		2FPS DSA		3FPS DSA		6FPS DSA		Single shot acquisition	
Reader	No shield	AGP shield	No shield	AGP shield	No shield	AGP shield	No shield	AGP shield	No shield	AGP shield
1	1.6	1.6	1.6	1.6	1.6	1.6	1.6	1.6	1.6	1.6
2	1.6	1.6	1.6	1.6	1.6	1.6	1.6	1.6	1.6	1.6
3	1.6	1.6	2.5	2.5	2.5	2.5	2.5	2.5	2.5	2.5
4	1.6	1.6	2	2	2	2	2	2	2	2
5	1	1	2	2	2	2	2	2	2	2
6	1.25	1.25	2	2	2	2	2	2	2	2
7	**1.25**	**2**	2	2	2	2	2	2	2	2
8	1.25	1.25	2	2	2	2	2	2	**2**	**1.6**
9	2	2	3	3	3	3	3	3	3	3
10	1.6	1.6	2	2	2	2	2	2	2	2
11	1.25	1.25	1.6	1.6	1.6	1.6	1.6	1.6	1.6	1.6
12	1.6	1.6	2	2	2	2	2	2	2	2

AGP, Aerosol Generating Procedure; DSA, digital subtraction angiography; FPS, frames per second.

Apart from one outlier reading each for reader 7 and 8, no difference in spatial resolution was observed with or without the shield by other readers.

**Figure 4.**
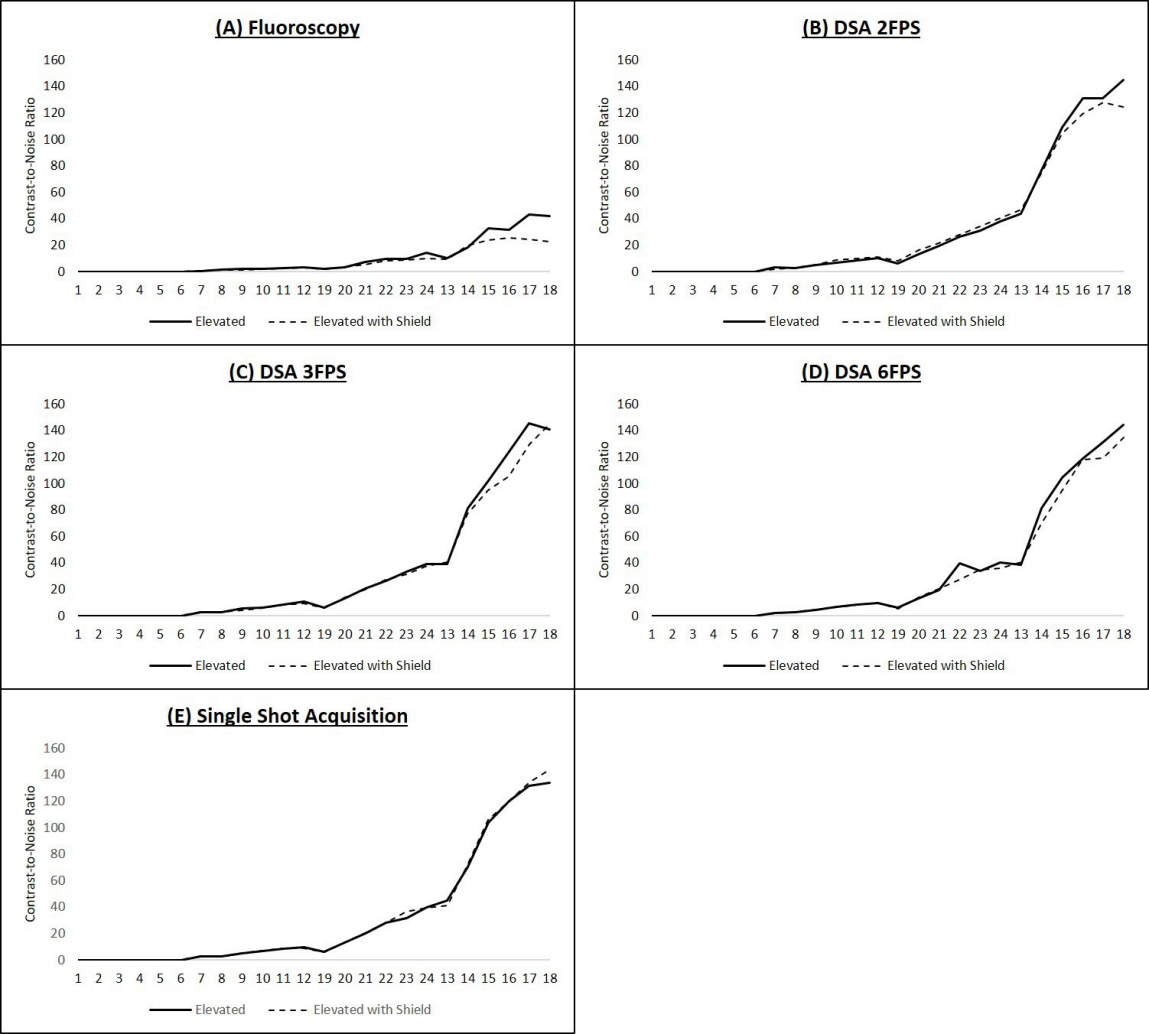
Contrast to noise ratios with and without the Shield on image quality test phantom. The CNR of positive contrast dots on an image quality test phantom was plotted for fluoroscopy (**A**), digital subtraction angiogram (DSA) at two frames per second (FPS) (**B**), DSA at 3 FPS (**C**), DSA at 6 FPS (**D**) and single shot acquisition (**E**). No statistical significance was demonstrated between the CNR with and without use of the Shield. DSA, digital subtraction angiography; FPS, frames per second.

Contrast dots 1–6 show negative contrast attenuation and were therefore not clinically relevant to our analysis. The CNR of the remaining 18 dots were plotted with the x-axis showing increasing contrast for the five exposure protocols both with and without the Shield (Figure 4). Each contrast column was evaluated using the *U*-test with and without the Shield. No statistical significance (*p* = 0.278, 0.361, 0.444, 0.444 for fluoroscopy, DSA 2FPS, DSA 3FPS, DSA 6FPS, and single shot acquisition respectively) was demonstrated with and without use of the Shield.

### Effect of SIR AGP Shield on radiation dose

The collected dosimetry, and dosemeter recorded kV and exposure time, are shown below in Table 2. The system dosimetry for the fluoroscopy exposures was not available as the Artis Zee system only shows the total K_a,r_ and P_KA_.

**Table 2.** Measured and reported radiation dosage with and without the Shield

Positioning and exposure protocols		kV	Time (s)	K_a,I_ (mGy) (Dosemeter)	K_a,r_ (mGy) (System)	P_KA_ (mGycm^2^) (System)
Fluoroscopy	Native	74	59.9	1.86	-	-
	Elevated	68	59.5	4.10	-	-
	Elevated with Shield	NR	59.4	4.49	-	-
DSA 2FPS	Native	69	20.3	7.23	13.3	5.88
	Elevated	67	20.3	22.9	25.9	7.99
	Elevated with Shield	68	20.3	28.9	32.3	9.98
DSA 3FPS	Native	69	20.0	11.01	20.3	8.95
	Elevated	68	20.0	40.0	44.5	13.7
	Elevated with Shield	68	20.1	43.8	48.8	15.1
DSA 6FPS	Native	69	20.0	22.2	40.9	18.0
	Elevated	71	20.0	95.3	103	31.9
	Elevated with Shield	71	20.0	107	117	36.0
Single Shot Acquisition	Native	72	0.0168	0.40	0.70	0.312
	Elevated	67	0.0641	0.59	0.70	0.207
	Elevated with Shield	68	0.0520	0.77	0.90	0.266

AGP, Aerosol Generating Procedure; DSA, digital subtraction angiography; FPS, frames per second.

Incident Air Kerma (K_a,I_) readings were obtained from the dosemeter while Air–Kerma at the Interventional Reference Point (K_a,r_) and Kerma–Air Product (P_KA_) were recorded from the Arits System.

A comparison of native and elevated position with shield demonstrated that the use of the Shield was associated with an increase in dose of 4.00, 3.98, 4.83, and 1.93 times for DSA 2FPS, DSA 3FPS, DSA 6FPS, and single shot acquisition respectively.

Comparing elevated positions with and without the shield demonstrates the effect of the AGP shield to the increase in dose of 1.26, 1.10, 1.13, and 1.30 times for DSA 2FPS, DSA 3FPS, DSA 6FPS, and single shot acquisition respectively.

## Discussion

The SIR AGP Shield has been evaluated by the UK Medicine and Healthcare products Regulatory Authority (MHRA) and the British Standards Institution (BSI), and is suitable for use as an adjunct to currently available personal protective equipment.

Existing Centers for Disease Control and Prevention (CDC) air exchange guidelines for negative pressure environment is 12 ACH for new rooms.^4^ Use of the Shield together with the hospital wall vacuum would provide a minimum equivalent rate of air exchange rate of a new negative pressure room to reduce chance of contamination to frontline workers.

Early models of barrier shields^5^ demonstrated their potential utility in reducing large droplet spread.^6^ Our human fluorescent droplet visualisation model allows for direct visual confirmation that the large droplets can be retained within the confines of the box reducing risk to operators on splash exposure of infectious droplets. While there have been many studies on cough and sneezing simulation for droplet spread especially in the context of COVID-19,^7–9^ as far as we are aware, this is the first time that droplet spread from coughing has been assessed from supine position. This is more physiologically representative of the working conditions of the interventional radiology theatre, where the greatest risk is likely to be from the patient coughing directly beside the operator during aerosol generating procedures. For smaller non-visible micro-aerosols generated during coughing, a flow dynamics study^10^ utilising the same barrier shield has estimated greater than 99% reduction of airborne particulates in the 1–500 µm range.

After 200 cycles of cleaning utilising commonly available hospital disinfectants, the material used for the shield did not demonstrate any significant drop in optical clarity. The maintenance of the optical clarity is important for visualisation of the airway structures during procedures.

Among the 60 paired subjective examinations of line-pair resolution, only one paired exam on acquisition was noted to have a one-step decrease in line-pair suggesting that the addition of the Shield is unlikely to have a significant impact on day-to-day fine resolution requirements. 10 out of 12 reviewers were also unable to discern any difference in spatial or contrast resolution with and without the use of the Shield. The minor differences observed by the two outlying observers may be of doubtful significance in routine use. There was no statistical evidence to show that overall CNR decreases with the use of the Shield.

The Shield itself does minimally contribute to the increase in radiation dose; however, the predominant cause of dose increase arises from the increased air gap between object and detector distance rather than the addition of the Shield. An option to reduce radiation dosage to patients would be to utilise the Shield for the initial intervention and subsequently switching to a smaller barrier shield like SIR HELMET^11^ to reduce radiation dose and increase ACH rates when access to airway is no longer required. For procedures involving the lower thorax, abdomen or lower limbs, the use of the barrier shield should not result in any clinical change of the positioning of the image intensifier and therefore should not result in any change in radiation dose.

Previous studies have documented extensive air, surface environmental and personal protective equipment contamination by symptomatic COVID-19 patients.^12^ By incorporating the use of a negative pressure barrier shield into routine practice on high-risk patients would provide an additional layer of protection to frontline healthcare workers against airborne/aerosolised pathogens and reduce environmental contamination. This would enable healthcare workers to perform their duties with greater peace of mind and reduced cognitive-load stress.
